# Estimating Unreported COVID-19 Cases with a Time-Varying SIR Regression Model

**DOI:** 10.3390/ijerph18031090

**Published:** 2021-01-26

**Authors:** Zhenghong Peng, Siya Ao, Lingbo Liu, Shuming Bao, Tao Hu, Hao Wu, Ru Wang

**Affiliations:** 1 Department of Graphics and Digital Technology, School of Urban Design, Wuhan University, Wuhan 430072, China; pengzhenghong@whu.edu.cn (Z.P.); aosiya@whu.edu.cn (S.A.); wh79@whu.edu.cn (H.W.); wang_ru@whu.edu.cn (R.W.); 2 Department of Urban Planning, School of Urban Design, Wuhan University, Wuhan 430072, China; 3 China Data Institute, Ann Arbor, MI 48108, USA; sbao@umich.edu; 4 Center for Geographic Analysis, Harvard University, Cambridge, MA 02138, USA; taohu@fas.harvard.edu

**Keywords:** SIR, time-varying parameters, unreported infection rate, infection fatality rate, COVID-19

## Abstract

Background: Potential unreported infection might impair and mislead policymaking for COVID-19, and the contemporary spread of COVID-19 varies in different counties of the United States. It is necessary to estimate the cases that might be underestimated based on county-level data, to take better countermeasures against COVID-19. We suggested taking time-varying Susceptible-Infected-Recovered (SIR) models with unreported infection rates (UIR) to estimate factual COVID-19 cases in the United States. Methods: Both the SIR model integrated with unreported infection rates (SIRu) of fixed-time effect and SIRu with time-varying parameters (tvSIRu) were applied to estimate and compare the values of transmission rate (TR), UIR, and infection fatality rate (IFR) based on US county-level COVID-19 data. Results: Based on the US county-level COVID-19 data from 22 January (T_1_) to 20 August (T_212_) in 2020, SIRu was first tested and verified by Ordinary Least Squares (OLS) regression. Further regression of SIRu at the county-level showed that the average values of TR, UIR, and IFR were 0.034%, 19.5%, and 0.51% respectively. The ranges of TR, UIR, and IFR for all states ranged from 0.007–0.157 (mean = 0.048), 7.31–185.6 (mean = 38.89), and 0.04–2.22% (mean = 0.22%). Among the time-varying TR equations, the power function showed better fitness, which indicated a decline in TR decreasing from 227.58 (T_1_) to 0.022 (T_212_). The general equation of tvSIRu showed that both the UIR and IFR were gradually increasing, wherein, the estimated value of UIR was 9.1 (95%CI 5.7–14.0) and IFR was 0.70% (95%CI 0.52–0.95%) at T_212_. Interpretation: Despite the declining trend in TR and IFR, the UIR of COVID-19 in the United States is still on the rise, which, it was assumed would decrease with sufficient tests or improved countersues. The US medical system might be largely affected by severe cases amidst a rapid spread of COVID-19.

## 1. Introduction

Although COVID-19 was reported several months ago [1], the coronavirus is still raging on a global scale, and is especially surging in the United States, which is one of the most important engines of the global economic network. The pandemic in the United States will have an important impact on the global economy and politics. It is fundamental to make relatively accurate estimates for preventing and controlling the COVID-19 pandemic in the United States [2,3], wherein the transmission rate (TR) and infection fatality rate (IFR) are key indicators [4].

The main obstacle to calculating such indicators is the unreported infection rate (UIR), which might be caused by insufficient testing, data depression of mild or asymptomatic patients, and a time-lag bias [5,6]. Direct use of IFR values derived from official data might lead to larger errors [7]. Similar research on SARS pointed out that preferential ascertainment of severe cases and delayed reporting of deaths are the main two reasons for case fatality risk (CFR) error [8]. Beyond insufficient early testing, mild and asymptomatic patients might cause most unreported cases. In Brazil, only some moderate and severe infectives in hospitalizations are recorded thus far [9]. On the other hand, the time lag deviation could be explained by the incubation period of COVID-19, which fluctuates in a wide range [10] and still possess a high transmittance [11]. The incubation period is also correlated to the age of the infectives, which can directly affect IFR [12]. It was concluded that the unreported cases might lead to four kinds of uncertainty in IFR calibration, with the unclear denominator, unknown infection time, unknown incubation, and undiagnosed asymptomatic infections [13].

Characterizing unreported cases has become a popular question in the epidemic modeling of COVID-19. The recent literature attempts to calculate the UIR or the reported rate (RR) based on country-level data [14,15,16], wherein, a single country-level data might lead to a greater bias [17]. Moreover, the county-level data in the United States on recovered infectives are not released. Thus, the calculation of IFR depends merely on the national aggregate data, which might further amplify the error. More and more studies use multinational data [18], county-level data [19], or country-county mixed regional data [20] for analysis, which greatly improves the accuracy of modeling by increasing the dimensionality and quantity of data.

However, previous studies seldom investigated the time effect of UIR, which might affect the accuracy of all indicators. A recent study suggested using a time-varying SIR model to capture the changing transmissive rate [21]. Moreover, the incubation period was shown to change in different stages of transmission [22]. Some studies showed that the possible value of COVID-19 IFR of China should be 2.3% [23], while another study showed that the early COVID-19 IFR in Wuhan might be as high as 20% [24]. Such disputes might also imply a changing trend in IFR.

This study proposes an SIR regression model with an unreported infection rate (SIRu) and SIRu, with time-varying parameters (tvSIRu) to estimate the values of TR, UIR, and IFR, and assess the impact of the time effect. The US county-level data used in this study comes from the open-source data of Johan Hopkins University on GitHub [25]. This study provides the first insights into the time series values of TR, UIR, and IFR of COVID-19, contributing to a deeper understanding of the trend of COVID-19 in the United States.

## 2. Materials and Methods

### 2.1. Data Source

The COVID-19 data used in this article contained 3142 counties in the United States, which included the number of daily new infectives, cumulative infectives, and deaths, while the population of recovered infectives remained unreported.

The date of the data ranged from 22 January 2020 to 20 August 2020, which contained 666,104 (3142 × 212) records. As a time-lag order (*t_k_*, *t_k+_*_1_) was applied in the data analysis, the number of whole records used for regression was 662,962 (3142 × 211).

### 2.2. tvSIRu Model with Fixed UIR

In the classic SIR dynamic model, the number of daily infectives ($I_{d}{}^{t_{k+1}}$) at time t_1_ could be expressed by the function of the infection rate *β*, the number of susceptible persons ($S^{t_{k}}$), infected persons ($I^{t_{k}}$), and the total population (*N*) at time *t_k_* (Equation (1)).

(1) $$I_{d}{}^{t_{k+1}}=\;\frac{\beta S^{t_{k}}I^{t_{k}}}{N}$$

**Figure 1:**
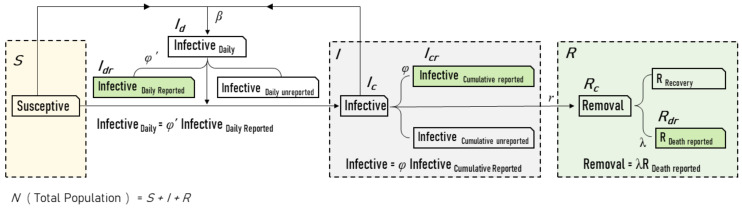
Susceptible–Infected–Recovered (SIR) model with unreported infection cases. The three big dashed boxes represent typical cabin parameters in the SIR model, wherein the infection data could be divided into two parts—reported and unreported. The solid green boxes represent the official released daily data on new infections, cumulative infections, and deaths, and might not represent the actual data on COVID-19 infection. Three new parameters were introduced to bridge such type of data suppression problem: φ’ is the unreported infection rate (UIR) of newly reported infections, φ is the UIR of cumulative reported infections, and λ represents the recovery/mortality rate of reported deaths (RDR).

The SIR model with unreported infection rate (SIRu) could be illustrated in Figure 1.

As the population of the recovered infectives was not released, two kinds of parameters were added to the SIR model, *λ* for the recovery/death rate (RDR), *φ* and *φ*′ for the unreported infection rate (UIR) of cumulative cases and daily cases, respectively. Such variables could be described as the following equations:

(2) $$I_{c}{}^{t_{k}}=\;\varphi I_{cr}{}^{t_{k}},\;R_{c}{}^{t_{k}}=\lambda R_{dr}{}^{t_{k}},\;I_{d}{}^{t_{k+1}}=\;\varphi^{\prime }I_{dr}{}^{t_{k+1}}$$

where $I_{c}{}^{t_{k}}$ represented the total cumulative infectives at time *t_k_*, and $I_{cr}{}^{t_{k}}$ denoted the cumulative cases reported. $R_{c}{}^{t_{k}}$ reflected the whole population of removals at time *t_k_*_,_ $R_{dr}{}^{t_{k}}$ as the cumulative death reported. The daily new infectives at time *t_k+_*_1_ ($I_{d}{}^{t_{k+1}}$) was calculated by *φ*′ and the corresponding data were reported ($I_{dr}{}^{t_{k+1}}$).

RDR could also be transformed into the infection fatality rate (IFR):

IFR = 1/(*λ* + 1) (3)

while considering a fixed UIR with no time effect, the UIR of total cumulative infectives and daily new cases could be considered equivalent, thus:

(4) $$\varphi =\varphi^{\prime }$$

The two explanatory variables in Equation (1), $S^{t_{k}}$, $I^{t_{k}}$, could be calculated as

(5) $$S^{t_{k}}=N-\;I_{c}{}^{t_{k}},\;I^{t_{k}}=I_{c}{}^{t_{k}}-\;R_{c}{}^{t_{k}}$$

The SIR model (Equation (1)) could be developed into Equation (6) by substituting Equations (2)–(4).

(6) $$\varphi I_{dr}{}^{t_{k+1}}=\frac{\beta \left(N-\varphi I_{cr}{}^{t_{k}}\right)\left(\varphi I_{cr}{}^{t_{k}}-\lambda \;R_{dr}{}^{t_{k}}\right)}{N}$$

Through further simplification and operation, Equation (6) could be transformed into Equation (7), which could be taken as the general tvSIRu model:

(7) $$I_{dr}{}^{t_{k+1}}=\;\beta I_{cr}{}^{t_{k}}-\frac{\beta \lambda }{\varphi }R_{dr}{}^{t_{k}}-\beta \varphi \frac{{\left(I_{cr}{}^{t_{k}}\right)}^{2}}{N}+\beta \lambda \frac{I_{cr}{}^{t_{k}}R_{dr}{}^{t_{k}}}{N}$$

Since the four combined variables, $I_{cr}{}^{t_{k}}$, $R_{dr}{}^{t_{k}}$, $\frac{{\left(I_{cr}{}^{t_{k}}\right)}^{2}}{N}$, $\frac{I_{cr}{}^{t_{k}}R_{dr}{}^{t_{k}}}{N}$, could be acquired or calculated by the data released, Equation (7) could be regarded as the primary linear function, Equation (8) with coefficients, *a*, *b*, *c*, *d*:

(8) $$I_{dr}{}^{t_{k+1}}=\;aI_{cr}{}^{t_{k}}+bR_{dr}{}^{t_{k}}+c\frac{{\left(I_{cr}{}^{t_{k}}\right)}^{2}}{N}+d\frac{I_{cr}{}^{t_{k}}R_{dr}{}^{t_{k}}}{N}$$

while considering the fixed-time effect of all three parameters in Equation (7), the corresponding average value (*β*, *λ*, *φ*) could be calculated in Equation (9).

(9) $$I_{dr}{}^{t_{k+1}}=\;\beta_{0}I_{cr}{}^{t_{k}}-\frac{\beta_{0}\lambda_{0}}{\varphi_{0}}R_{dr}{}^{t_{k}}-\beta_{0}\varphi_{0}\frac{{\left(I_{cr}{}^{t_{k}}\right)}^{2}}{N}+\beta_{0}\lambda_{0}\frac{I_{cr}{}^{t_{k}}R_{dr}{}^{t_{k}}}{N}$$

where the values of *β*_0_, *λ*_0_, *φ*_0_ are constants.

### 2.3. tvSIRu Model with Time-Varying UIR

If the UIR varied over time, the UIRs of the cumulative cases and daily new cases were different, which was defined as *φ* and *φ*′, respectively. Equation (6) could be rewritten as

(10) $$\varphi^{\prime }I_{dr}{}^{t_{k+1}}=\frac{\beta \left(N-\varphi I_{cr}{}^{t_{k}}\right)\left(\varphi I_{cr}{}^{t_{k}}-\lambda \;R_{dr}{}^{t_{k}}\right)}{N}$$

To simplify the computation, a new parameter *β*′ was introduced:

(11) $$\beta^{\prime }=\beta /\varphi^{\prime }$$

Then Equation (10) could be transformed into a similar form of Equation (7):

(12) $$I_{dr}{}^{t_{1}}=\;\beta^{\prime }I_{cr}{}^{t_{k}}-\frac{\beta^{\prime }\lambda }{\varphi }R_{dr}{}^{t_{k}}-\beta^{\prime }\varphi \frac{{\left(I_{cr}{}^{t_{k}}\right)}^{2}}{N}+\beta^{\prime }\lambda \frac{I_{cr}{}^{t_{k}}R_{dr}{}^{t_{k}}}{N}$$

To verify the assumption of time-varying parameters, the coefficients in Equations (7) and (12) could be represented by the initial values and time effect functions. Such functions were substituted into the two models gradually, resulting in several sub-equations with time effects.

(13) $$\beta =\;\beta_{0}g\left(t\right)$$

(14) $$\beta =\;\beta_{0}g\left(t\right),\;\lambda =\;\lambda_{0}h\left(t\right)$$

(15) $$\beta^{\prime }=\;{\beta^{\prime }}_{0}g^{\prime }\left(t\right),\;\lambda =\;\lambda_{0}h\left(t\right),\;\varphi =\;\varphi_{0}f\left(t\right)$$

Substituting Equations (13)–(15) into Equations (7) and (12), three complete equations could be generated:

(16) $$I_{dr}{}^{t_{1}}=\;\beta_{0}g\left(t\right)I_{cr}{}^{t_{k}}-\frac{\beta_{0}\lambda g\left(t\right)}{\varphi }R_{dr}{}^{t_{k}}-\beta_{0}g\left(t\right)\varphi \frac{{\left(I_{cr}{}^{t_{k}}\right)}^{2}}{N}+\beta_{0}g\left(t\right)\lambda \frac{I_{cr}{}^{t_{k}}R_{dr}{}^{t_{k}}}{N}$$

(17) $$I_{dr}{}^{t_{1}}=\;\beta_{0}g\left(t\right)I_{cr}{}^{t_{k}}-\frac{\beta_{0}g\left(t\right)\lambda_{0}h\left(t\right)}{\varphi }R_{dr}{}^{t_{k}}-\beta_{0}g\left(t\right)\varphi \frac{{\left(I_{cr}{}^{t_{k}}\right)}^{2}}{N}+\beta_{0}g\left(t\right)\lambda_{0}h\left(t\right)\frac{I_{cr}{}^{t_{k}}R_{dr}{}^{t_{k}}}{N}$$

(18) $$I_{dr}{}^{t_{1}}=\;{\beta^{\prime }}_{0}g^{\prime }(t)(\varphi_{0}f(t)I_{cr}{}^{t_{k}}-\lambda_{0}h\left(t\right)R_{dr}{}^{t_{k}}-\varphi_{0}{}^{2}f{\left(t\right)}^{2}\frac{{\left(I_{cr}{}^{t_{k}}\right)}^{2}}{N}+\lambda_{0}h\left(t\right)\varphi_{0}f\left(t\right)\frac{I_{cr}{}^{t_{k}}R_{dr}{}^{t_{k}}}{N})$$

In terms of the specific functions reflecting time effect, the power, exponential, and periodic function were tested and compared in this article:

(19) $$\tau_{1}\left(t\right)=x{\;}^{t},\;\tau_{2}\left(t\right)=t{\;}^{x},\;\tau_{3}\left(t\right)=\frac{1}{2}\left(1+\cos\left(\frac{t}{x}\pi \right)\right)$$

This study tested the five equations, (8), (9), (16), (17), and (18), where Equation (8) is the OLS linear regression derived from the SIRu model, Equation (9) is SIRu with fixed-time effect, Equations (16), (17), and (18) are tvSIRu with single time-varying β, time-varying β and λ, all time-varying parameters of β, λ, and φ, respectively.

## 3. Results

### 3.1. OLS and SIRu Regressions

The linear regression derived from the SIRu model showed acceptable fitness and the adjusted R^2^ was 0.4813 (n = 662,962) (Table 1). The negative value of coefficients b and c were consistent with the corresponding operation signs in Equation (7). Such results verified the assumption of the SIRu model to a certain extent.

The SIRu model with a fixed-time effect in Equation (9) further provided the estimated value of TR, UIR, and RDR (Table 2). The results showed that the average *β*_0_ value from 22 January to 20 August was 0.0339 (95%CI 0.0338–0.0340), and the φ_0_ value was 19.5 (95%CI 19.38–19.54), which implied that there might be 19.5 undiagnosed cases while one infection was reported in US counties, on average. Meanwhile, the *λ*_0_ value of 192.5 (95%CI 191.790–193.243) could be interpreted as an IFR value of 0.516%.

### 3.2. SIRu at the State Level

**Figure 2:**
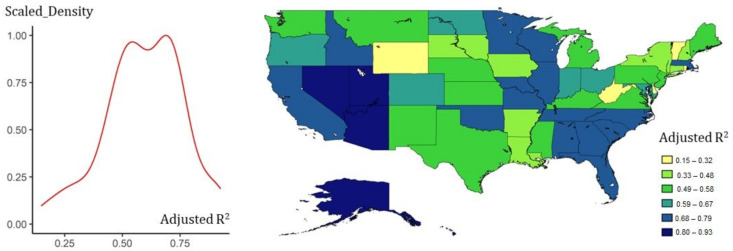
State-level fitness of Equation (8) with county-level data. The scaled density curve of adjusted R^2^ shows that Equation (8) was generally applicable, and its mapping indicated that the potential spatial heterogeneity of the states would affect the results of the SIRu modeling. Among them, the states in the southeastern, the west coast, and the Great Lakes Region showed higher adaptability.

**Figure 3:**
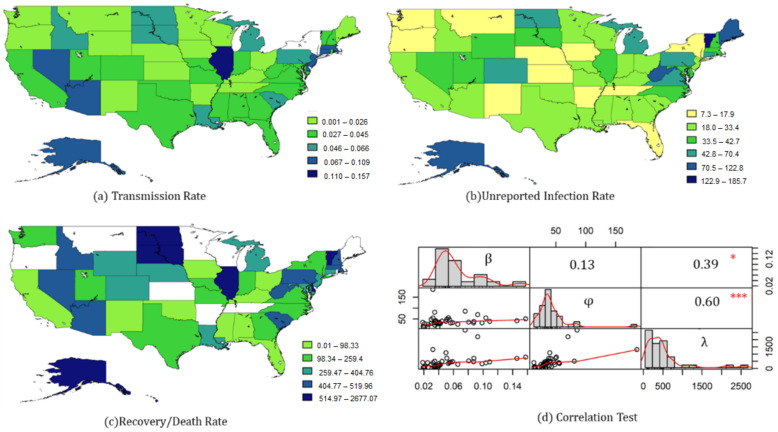
State-level parameters of Equation (9) with county-level data. (**a**) Transmission rate—three obvious clusters could be identified, Nevada–Arizona, Illinois, and Massachusetts–New Jersey, wherein the coefficient of New York could not be applied due to the non-significant *p*-value. (**b**) Unreported infection Rate. The UIR in the northeast was relatively high, but there were also two central states with high values. (**c**) Recovery/Death Rate. Blank blocks indicate that the RDR in the area was not applicable due to the insignificant *p*-value, wherein, the RDR of the northeastern cities was relatively higher, while the west coast states had both a high TR and RDR. (**d**) Correlation Test. The Pearson correlation test of all states’ parameters with significant *p*-values showed an obvious connection between RDR and TR, UIR. Note: *: significant at 0.05 level; ***: significant at 0.001 level.

The study further utilized county-level data to compare state-level parameters based on fixed-time effects. Figure 2 shows the fitness of Equation (8) across the whole states, most of which were above 0.5 (Figure 2), and each state had different TR, UIR, and RDR values in Equation (9), which indicated an obvious spatial heterogeneity in the transmission of COVID-19 (Figure 3). All parameters and statistical descriptions are reported in Appendix A, Appendix B and Appendix C.

Most states had a TR between 0.018–0.053, seven states with relatively high values were Illinois (0.146), Massachusetts (0.109), Connecticut (0.104), New Jersey (0.098), Nevada (0.080), Arizona (0.087), and Alaska (0.076) (Figure 3a).

In terms of UIR, most states were concentrated between 28–50 (Figure 3b). Some states had relatively lower values, such as New York (7.31) and Oregon (8.64), while the top five states were Maine (122.84), Vermont (185.66), Alaska (85.69), and West Virginia (80.90).

The fitting results on RDR in some cities were not significant, but most significant values were between 200–500, which was equivalent to the value of IFR ranging from 0.2% to 0.5% (Figure 3c). Wherein, eight cities were reported below 99 (IFR > 1%), including Ohio (44.29), Oklahoma (49.35), Florida (77.97), Alabama (66.40), Mississippi (98.32), Kentucky (74.40), Iowa (58.45), New Mexico (62.18), and California (55.91).

The Pearson correlation between the three state-level indicators was also tested, showing a positive correlation between UIR and RDR. In other words, the lower the IFR, the higher the UIR (Figure 3d).

### 3.3. tvSIRu Regression at the Country Level

**Figure 4:**
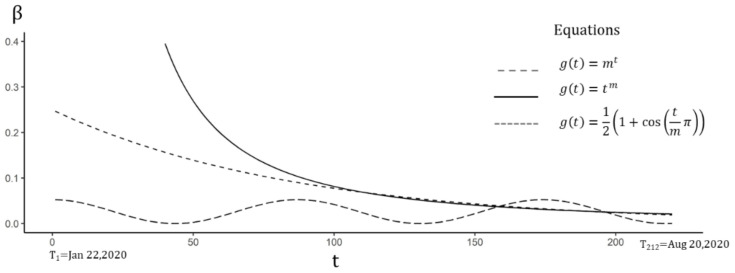
Time-varying TR estimated by Equation (16). Although the initial values of the power function were much higher than the exponential function in the medium term, the two values tended to be the same, while the periodic function showed that it was in the third wave.

The tvSIRu model with time-varying TR was first tested by three sub-equations of Equation (16), and the AIC of all equations was reduced, by comparing to the SIRu model of fixed-time effect (Table 3). Meanwhile, all estimated TR displayed a declining trend (Figure 4). Wherein, the power function showed the best fitness with an initial extremely high value of 227.58 (95%CI 219.89–235.27) decreasing to 0.022 on 20 August. Such a high value might reflect the high contagiousness of COVID-19 in the early stage. The corresponding UIR and RDR were 18.61 (95%CI 18.52–18.69) and 183.34 (95%CI 182.63–184.05), which were slightly higher than the values in Equation (9).

**Figure 5:**
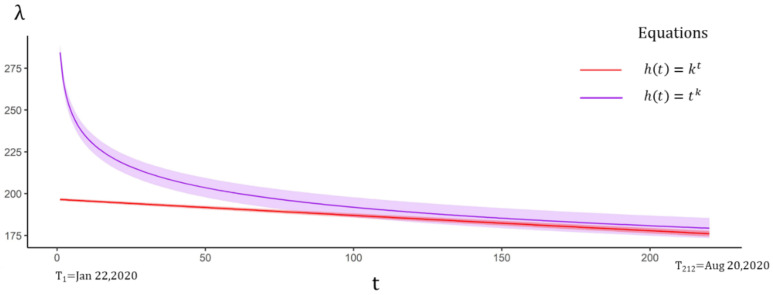
Time-varying RDR with 95% CI estimated by Equation (17). If the time effect of UIR was not considered, the fitting results showed that RDR exhibited a decreasing effect over time, which meant that IFR might be slowly increasing.

When the time effect of RDR was further added to Equation (17), the AIC of the power function displayed a slight decrease in Equation (17) (Table 4). Wherein, the UIR was 19.02 (95%CI 18.93–19.12), which was similar to the value in Equation (9). However, both equations showed decreasing trends in the changing RDR, implying an increase of IFR (Figure 5).

**Figure 6:**
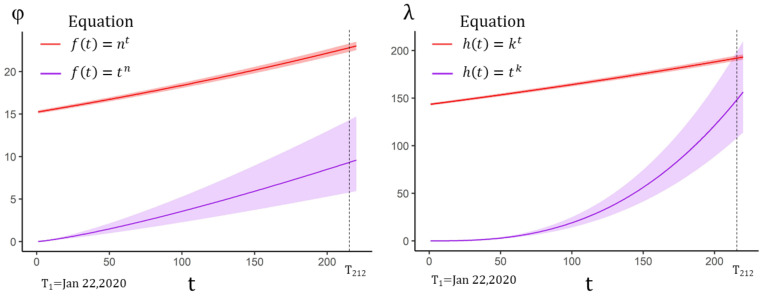
Time-varying UIR and RDR with 95%CI estimated by Equation (18). Equation (18) only provided the estimated values of UIR and RDR. Both the power function and the exponential function implied an increasing effect, wherein, the power function was much smaller than the exponential function in terms of UIR estimation.

The power function also showed better performance in tvSIRu with all three time-varying parameters estimated by Equation (18), which indicated a gradual increase in both UIR and RDR (Table 5). This trend indicated that the initial UIR and RDR were relatively low (Figure 6). The value of UIR and RDR achieved 9.1 (95%CI 5.7–14.0) and 141.706 (95%CI 103.3358–189.9486) at T_212_ on 20 August, respectively. IFR could be calculated as 0.70% (95%CI 0.52–0.95%). Based on the officially released data on 20 August 2020, it might be concluded that about 30% of the whole population was infected. 

## 4. Discussion

Few studies analyzed the time-varying UIR of COVID-19, and its impacted on the estimation of TR and IFR. This study estimated the values of UIR, TR, and IFR of both time-fixed effect and time-varying effect with tvSIRu models, based on county-level data.

In terms of the fixed-time effect, the results showed that from 22 January to 20 August, the average TR and UIR at the country level in the United States were 0.03 and 19.5, respectively, and the RDR was 192.5, which also meant that the IFR was 0.516%. The IFR was slightly lower than the overall IFR of 0.66% estimated in China [17], while the CDC in the United States recommends 0.65% [26].

In a further analysis on the state level, the UIR of all states ranged from 7.32–185.66 (mean = 38), and the IFR ranged from 0.037–2.20% (mean = 0.21%). A related study on 20 US counties estimated that the range of UIR was 4.32–776.68 (mean = 27.7) and IFR was 0.02–1.81% (mean = 0.027%), the range of UIR estimated by the SIRu model was more concentrated, and the IFRs had a similar upper boundary [27]. Another previous study estimated four states’ upper boundary of UIR—Illinois (40.86), Massachusetts (38.28), New Jersey (29.22), and New York (35.17) [19]. Among these, the first three were similar to the values estimated by the SIRu model, which were 41.51, 39.22, and 31.83, only New York had a different value of 7.32. However, interestingly the study also pointed out that the UIR estimated by an antibody test in New York State in early May was around 7.6, which might indicate the stability of the SIRu method.

Based on the tvSIRu model, UIR and IFR increased by following the power function rather than the exponent function, which was the default setting in previous research [21]. Other than the average value of 0.03 in SIRu, the TR estimated by the tvSIRu model decreased from a large value of 227 to a value of 0.022 on 20 August, which was much lower than the fixed value 0.05–0.06 reported in related research [21]. It might further explain the high contagiousness in the initial stage in COVID-19 transmission. The increasing UIR estimated by the tvSIRu model had a similar value of 9.1 (95%CI 5.7–14.0) at T_212_ (20 August), which was very close to the value of 9 estimated in a former study in April [20], and the latest study in September [28]. The UIR value was also close to the value reported in Brazil (Reported rate = 9.2%, UIR = 10.8) [18]. Such similarity in the estimated UIR in different periods might be caused by the fixed-time effect in the former models, which only represented the average values of UIR, as calculated by the SIRu model. The increasing UIR meant that the IFR was on a downward trend. The value of IFR on August 20 was 0.70% (95%CI 0.52–0.95%), which was still close to the value recommend by CDC [26].

Many studies supposed that the UIR would decrease with the improvement of COVID-19 testing and increased hygiene awareness, but our research showed that UIR in the United States is increasing, which might have a great impact on policy-making for COVID-19 prevention. On the other hand, empirical TR is often used in contemporary COVID-19 modeling, but the tvSIRu model indicates that the COVID-19 infection rate changed dramatically. The initial value of TR was 246, reflecting that this pandemic was extremely contagious in the early transmission stage of the United States. Previous SIR modeling seldom characterized such a feature, which might lead to large estimation errors. The reducing TR, IFR, and increasing UIR indicated by the model showed that the epidemic was rapidly spreading in the United States with a large number of self-healing populations. However, it is noteworthy the potential increasing cases of severe illnesses might greatly affect the medical system, and the relevant departments still need to provide more protection to high-risk groups.

As shown in Figure 3, with the potential pattern of spatial correlation, the tvSIRu model could be developed by integrating models considering the spatial weight, to detect the spatiotemporal features of COVID-19 transmission, such as Geographical Weighed Regression model (GWR) [29], Spatial Panel Model, etc. Meanwhile, the regression used in the tvSIRu models could also be extended by a non-linear method, such as the Artificial Neural Network (ANN) [30].

## 5. Conclusions

This article indicates that there might be an increasing number of unrecorded COVID-19 cases in the official U.S. data, wherein, the tvSIRu model provides a simple, convenient, and relatively accurate calculation of the unreported parameters of COVID-19 with time effect, based on official released data. Moreover, this method can be easily transplanted to analyze the epidemic modeling of other countries.

It must be admitted that if single level geography units of data are used, the independent variables might display strong collinearity, leading to overfitting. It is therefore necessary to use proper sub-geographical level data to fit the national-level or state-level data. Furthermore, the non-linear model regression was based on the Gauss-Newton iteration, which could be further optimized with machine learning models.

## Figures and Tables

**Table 1:** Linear SIR Regression estimated by Equation (8).

	Estimate	Std. Error	*t* Value	*p*-Value	*Significance*
Intercept	0.9445	0.0617	15.29	<0.001	***
*a*	0.0283	0.0001	421.44	<0.001	***
*b*	−0.1853	0.0011	−161.50	<0.001	***
*c*	−0.5392	0.0023	−227.86	<0.001	***
*d*	4.6718	0.0241	193.40	<0.001	***
Adjusted R^2^	0.4813			<0.001	***
AIC	7,059,288				

Note: AIC: model fitness based on Akaike information criterion; ***: significant at 0.001 level.

**Table 2:** SIR Regression estimated by Equation (9).

	Estimate	Std. Error	*t* Value	*p*-Value
*β*_0_	0.0339	0.0001	604.4	*<0.001*
*φ*_0_	19.4603	0.0415	468.7	*<0.001*
*λ*_0_	192.5163	0.3707	519.3	*<0.001*
AIC	7,080,522			

Note: *β*_0_: the average transmission rate; *φ*_0_: the average unreported infection rate; λ**_0_: the average recovery/mortality rate of reported deaths; AIC: Akaike information criterion.

**Table 3:** Time-varying TR estimated by Equation (16).

	$g\left(t\right)=m^{t}\;$		$g\left(t\right)=t^{m}\;$		$g\left(t\right)=\frac{1}{2}\left(1+\cos\left(\frac{t}{m}\pi \right)\right)\;$	
	Estimate	*p*-Value	Estimate	*p*-Value	Estimate	*p*-Value
*β*_0_	0.2498	<0.001	227.5862	*<0.001*	0.0525	<0.001
*φ*	18.5069	<0.001	18.6100	*<0.001*	19.7915	<0.001
*λ*	181.9526	<0.001	183.3437	*<0.001*	196.0005	<0.001
*m*	0.9883	<0.001	−1.7229	*<0.001*	43.5300	<0.001
*AIC*	6,982,233		6,962,783		7,076,624	

Note: *β*_0_: the initial constant in the function of time-varying transmission rate; *φ:* the unreported infection rate; *λ*: the recovery/mortality rate of reported deaths; *m*: the estimated constant in power/exponential function of the time variable; AIC: model fitness based on Akaike information criterion.

**Table 4:** Time-varying TR and RDR estimated by Equation (17).

	$g\left(t\right)=m^{t}$ $,\;h\left(t\right)=k^{t}$		$g\left(t\right)=t^{m},\;\;h\left(t\right)=t^{k}$	
	Estimate	*p*-Value	Estimate	*p*-Value
*β*_0_	0.25239807	<0.001	241.912633	<0.001
*φ*	18.66057736	<0.001	19.024919	<0.001
*λ*_0_	196.63534702	<0.001	284.386081	<0.001
*m*	0.98828594	<0.001	−1.734960	<0.001
*k*	0.99949887	<0.001	−0.085439	<0.001
*AIC*	6,980,073		6,959,144	

Note: *β*_0_: the initial constant in the function of time-varying transmission rate; *φ:* the average unreported infection rate; *λ*_0_: the initial constant in the function of time-varying recovery/mortality rate; *m,k*: the estimated constant in power/exponential functions of the time variable; AIC: model fitness based on Akaike information criterion.

**Table 5:** Time-varying UIR and RDR estimated by Equation (18).

	$g\left(t\right)=m^{t}$ $,\;f\left(t\right)=n^{t}$ $,\;h\left(t\right)=k^{t}(E.1)$		$g\left(t\right)=t^{m}$ $,\;f\left(t\right)=t^{n}$ $,\;h\left(t\right)=t^{k}(E.2)$	
	Estimate	*p*-Value	Estimate	*p*-Value
*β* *′*_0_	0.2507	<0.001	40.1660	<0.001
*φ*_0_	15.2287	<0.001	0.0109	<0.001
*λ*_0_	143.4179	<0.001	0.0001	<0.001
*m*	0.9838	<0.001	−2.6890	<0.001
*n*	1.0018	<0.001	1.2555	<0.001
*k*	1.0013	<0.001	2.6687	<0.001
*AIC*	6,969,888		6,813,832	

Note: *β*’**_0_: the initial constant in the time-varying function of the transmission rate and the unreported rate of new reported infections; *φ*_0_: the initial constant in the time-varying function of the unreported rate of cumulative reported infections; *λ*_0_: the initial constant in the function of time-varying recovery/mortality rate; *m,n,k*: the estimated constants in power/exponential functions of the time variable; AIC: model fitness based on Akaike information criterion.

## Data Availability

The data used in this study comes from the open-source data of Johan Hopkins University on GitHub (https://github.com/CSSEGISandData/COVID-19).

## Appendix A

**Table A1:** Parameters Estimated by Equation (7) on State Level.

State	R^2^	β (TR)	φ (UIR)	λ (RDR)	IFR
Alabama	0.6800	0.0384	28.1118	66.4050	0.0148
Alaska	0.7043	0.0758	85.6993	2677.0740	0.0004
Arizona	0.8344	0.0873	31.8510	459.3959	0.0022
Arkansas	0.4323	0.0202	14.4224	−103.5099 *	
California	0.7199	0.0337	31.9797	55.9131	0.0176
Colorado	0.6327	0.0324	55.2171	395.2257	0.0025
Connecticut	0.4209	0.1039	55.4896	389.6164	0.0026
Delaware	0.3171	0.0875	38.4571	708.5320	0.0014
District of Columbia	0.7049	0.1575	50.9941	780.3851	0.0013
Florida	0.7076	0.0391	13.4101	79.9765	0.0123
Georgia	0.7657	0.0402	28.1156	195.6581	0.0051
Hawaii	0.8935	0.0928	70.3956	2208.2030	0.0005
Idaho	0.7048	0.0588	33.2108	466.1497	0.0021
Illinois	0.7909	0.1457	41.5079	693.7160	0.0014
Indiana	0.6182	0.0301	29.8719	255.2491	0.0039
Iowa	0.4786	0.0190	15.7639	58.4500	0.0168
Kansas	0.5360	0.0230	16.0095	−81.6163 *	
Kentucky	0.5781	0.0259	25.9078	74.4067	0.0133
Louisiana	0.4316	0.0658	25.3544	284.9328	0.0035
Maine	0.5311	0.0223	122.8401	−543.0694 *	
Maryland	0.6333	0.0406	35.1383	325.5810	0.0031
Massachusetts	0.6904	0.1089	39.2230	448.5265	0.0022
Michigan	0.4950	0.0533	56.0760	339.5087	0.0029
Minnesota	0.7441	0.0068	30.1427	−1098.4030 *	
Mississippi	0.5179	0.0349	21.9778	98.3266	0.0101
Missouri	0.7096	0.0412	31.3774	188.5405	0.0053
Montana	0.5230	0.0229	11.8879	−139.4672 *	
Nebraska	0.5644	0.0250	13.2247	400.8106	0.0025
Nevada	0.9250	0.0806	35.9869	514.9621	0.0019
New Hampshire	0.4695	0.0446	40.4871	486.1162	0.0021
New Jersey	0.5094	0.0986	31.8312	323.4239	0.0031
New Mexico	0.5075	0.0197	13.4675	62.1788	0.0158
New York	0.4734	−0.0221 *	7.3187	244.7202	0.0041
North Carolina	0.7307	0.0355	39.0629	182.6843	0.0054
North Dakota	0.6131	0.0511	49.7167	715.7587	0.0014
Ohio	0.6007	0.0213	28.9136	46.3505	0.0211
Oklahoma	0.7452	0.0362	33.4416	44.2904	0.0221
Oregon	0.6663	0.0166	8.6465	−24.6175 *	
Pennsylvania	0.5268	0.0547	47.0019	407.8571	0.0024
Rhode Island	0.2214	0.0373	40.5705	−56,191.3200 *	
South Carolina	0.6950	0.0563	31.2758	433.8097	0.0023
South Dakota	0.4069	0.0500	39.8930	1084.9280	0.0009
Tennessee	0.6854	0.0200	17.9252	−388.7768 *	
Texas	0.5605	0.0424	28.6138	250.5731	0.0040
Utah	0.9043	0.0361	42.7415	119.1223	0.0083
Vermont	0.1490	0.0322	185.6625	1314.2170	0.0008
Virginia	0.5568	0.0299	48.3013	259.4586	0.0038
Washington	0.5531	0.0213	15.1840	115.7273	0.0086
West Virginia	0.3163	0.0346	80.9098	497.3075	0.0020
Wisconsin	0.7368	0.0206	28.0754	−231.9747 *	
Wyoming	0.2622	0.0185	34.7665	404.7622	0.0025

* *p* value > 0.05.

## Appendix B

**Table A2:** Statistical Prescription of State Level Parameters on COVID-19.

	Min.	1st Qu.	Median	Mean	3rd Qu.	Max.
TR	0.006824	0.0235	0.036772	0.047826	0.055902	0.157463
UIR	7.318659	25.63112	31.9797	38.89121	42.12469	185.6625
RDR	44.29042	135.0128	332.5448	456.1626	481.1246	2677.074

Note: TR: the transmission rate; UIR: the unreported infection rate; RDR: the recovery/mortality rate.

## Appendix C

**Table A3:** Parameters Table List.

Parameters	References
β	the transmission rate of COVID-19 in SIR model
φ′	the unreported infection rate of new reported infections
*φ*	the unreported infection rate of cumulative reported infections
λ	the recovery/mortality rate of reported deaths
$I_{c}{}^{t_{k}}$	the total cumulative infectives at time *t_k_*
$I_{cr}{}^{t_{k}}$	the cumulative cases reported at time *t_k_*
$R_{c}{}^{t_{k}}$	the whole population of removals at time *t_k_*
$R_{dr}{}^{t_{k}}$	the cumulative death reported at time *t_k_*
$I_{d}{}^{t_{k+1}}$	the factual daily new infectives at time *t_k+_*_1_
$I_{dr}{}^{t_{k+1}}$	the reported daily new infectives at time *t_k+_*_1_
$S^{t_{k}}$	the number of susceptible persons at time *t_k_*
$I^{t_{k}}$	the number of infectives at time *t_k_*
